# Proxies of energy expenditure for marine mammals: an experimental test of “the time trap”

**DOI:** 10.1038/s41598-017-11576-4

**Published:** 2017-09-18

**Authors:** Monique A. Ladds, David A. S. Rosen, David J. Slip, Robert G. Harcourt

**Affiliations:** 1 School of Mathematics and Statistics, Victoria University of Wellington, Wellington, 6012 New Zealand; 2 Marine Predator Research Group, Department of Biological Sciences, Macquarie University, North Ryde, 2113 NSW Australia; 3 Marine Mammal Research Unit, Institute for the Oceans and Fisheries, University of British Columbia, 2202 Main Mall, Vancouver, BC V6T 1Z4 Canada; 4 Taronga Conservation Society Australia, Bradley’s Head Road, Mosman, 2088 NSW Australia

## Abstract

Direct measures of energy expenditure are difficult to obtain in marine mammals, and accelerometry may be a useful proxy. Recently its utility has been questioned as some analyses derived their measure of activity level by calculating the sum of accelerometry-based values and then comparing this summation to summed (total) energy expenditure (the so-called “time trap”). To test this hypothesis, we measured oxygen consumption of captive fur seals and sea lions wearing accelerometers during submerged swimming and calculated total and rate of energy expenditure. We compared these values with two potential proxies of energy expenditure derived from accelerometry data: flipper strokes and dynamic body acceleration (DBA). Total number of strokes, total DBA, and submergence time all predicted total oxygen consumption $({\boldsymbol{sV}}{{\boldsymbol{O}}}_{{\boldsymbol{2}}}$ ml kg^−1^). However, both total DBA and total number of strokes were correlated with submergence time. Neither stroke rate nor mean DBA could predict the rate of oxygen consumption ($s\mathop{{\boldsymbol{V}}}\limits^{{\boldsymbol{.}}}{{\boldsymbol{O}}}_{{\boldsymbol{2}}}$ ml min^−1^ kg^−1^). The relationship of total DBA and total strokes with total oxygen consumption is apparently a result of introducing a constant (time) into both sides of the relationship. This experimental evidence supports the conclusion that proxies derived from accelerometers cannot estimate the energy expenditure of marine mammals.

## Introduction

Two primary components of the energy expended to acquire prey for marine mammals are the cost of travelling to the foraging destination and the energy expended from diving, hunting, and capturing prey^1^. As air-breathing vertebrates, marine mammals face unique challenges as their prey is patchily distributed throughout the ocean, often in deep water^2^. This requires swimming long distances and diving to great depth in ocean waters which entails significant energy expenditure^3,4^. Measuring this energy expenditure is most accurately done with respirometry systems that measure oxygen consumption, but this method is essentially confined to the laboratory^5–7^. Therefore, alternative methods are required to measure the energy expenditure of marine mammals in the field.

Recent approaches for estimating energy expenditure include monitoring heart rate (reviewed in Green^8^) and measuring turnover of doubly labelled water (DLW) (reviewed in Butler, *et al*.^9^). However, both methods suffer from various issues, such as cost, level of invasiveness, and accuracy. Accelerometry, from which other proxies of energy consumption can be derived, offers an affordable, less invasive, and potentially more reliable alternative^10–12^. Data obtained from small accelerometers affixed to the animal can be used to predict stroke rate^3^ or a derivative of dynamic body acceleration (DBA), expressed either as vectorial (VeDBA) or as an overall measure (ODBA)^13^. ODBA and VeDBA are calculated from body acceleration measured on three axes^14^, while stroke rate can be calculated from the peaks in the dynamic acceleration of the x-axis^15,16^.

While these methods have demonstrated strong predictive relationships to oxygen consumption in terrestrial animals^17^, the results in marine mammals and birds have so far been mixed^18–20^. The number of strokes was shown to be useful in predicting energy consumption in Weddell seals (*Leptonychotes weddellii*)^3^, northern fur seals (*Callorhinus ursinus*), and Antarctic fur seals (*Arctocephalus gazella*)^21^. Similarly, in a sample of Steller sea lions (*Eumetopias jubatus*) swimming to feeding tubes at depth, activity (measured by ODBA) correlated well with oxygen consumption (measured by respirometry^11^); albeit, this was with a small effect size (see Halsey, *et al*.^14^ for a rebuttal). However, when data were analysed within different dive types using the same animals there was no significant relationship between ODBA and active metabolic rate^22,23^. Similarly there was no relationship found with double-crested cormorants (*Phalacrocorax auritus*) when measuring oxygen uptake between dives^24^. Further, a poor relationship was found between ODBA and energy expenditure (measured via DLW) for northern fur seals, with the weakness attributed to trying to incorporate all activity into one measure^25^. When individual activities were identified (diving, transiting, or resting) a strong relationship was found between VeDBA and energy expenditure (as measured by DLW)^10^. Consistent with this relationship was recent research on imperial cormorants (*Phalacrocorax atriceps*) where mass-specific energy expenditure for resting, diving and walking were related to ODBA, although no relationship could be established for flying^20^.

Therefore it appears that the use of accelerometry data for measuring energy expenditure (either via stroke rate or a measure of DBA) is feasible; however, a recent commentary critiques this approach arguing that at least some of these relationships may have resulted from what has been termed the “time-trap”^26^. The time trap refers to conflating measurements that have time on both sides of the equation. For instance, if the relationship between metabolism and DBA is derived by correlating the sum of energy expenditure against the sum of accelerometer output (either number of strokes or DBA), then time is included in both the dependent and independent variables. Regressing these two values with one-another is likely to result in a strong relationship as time is correlated with itself^26^. Specifically, this occurs when the integral of the energy expenditure (in this case VO_2_) and the integral of the proxy (usually total number of strokes^3^ or DBA^10^) are calculated, resulting in a single point per dive for each animal. As dive duration increases, the number of strokes or the sum of the DBA also inevitably increases, such that the resulting model usually indicates a strong positive correlation. If the relationship is based solely on the contribution of “time” to both sides of the equation, it will disappear when both variables are presented as a rate (averaged by time); for example when mean VO_2_ is regressed against stroke rate or mean DBA^24^. However, if these relationships persist in the absence of time, then accelerometry could prove an important tool for predicting energetic expenditure. Here we experimentally test the effects of time on the relationship of energy expenditure (as measured via respirometry) both with stroke rate and DBA in a controlled laboratory environment.

Further, in a recent study on the derivation of stroke rate we revealed that the parameters used to estimate stroke rate had a significant effect on overall accuracy^27^. Based on these analyses, we surmised that the parameters used to calculate DBA would also affect how accurately DBA predicts energy. Therefore, in this study we also test whether logger positioning, attachment method, and the subsequent calculations to derive DBA, influence its ability to predict energy expenditure.

## Results

### Rates of oxygen consumption

Animals completed between 7 and 35 trials, defined as one submerged swim with a complete recovery (return to baseline levels of oxygen consumption). Submergence times ranged from 26 to 221 sec (Table 1), and larger animals on average remained submerged for longer than smaller animals (Table 1). $sV{O}_{2}$ ranged from 5.44 to 115.00 (ml kg^−1^) and $s\dot{V}{O}_{2}$ ranged from 6.49 to 41.67 (ml min^−1^ kg^−1^).

**Table 1:** Seal characteristics, accelerometer details and summary metabolic rates from all trials.

Species	ID	Mass	Age	Marine facility	Device	Recording rate	Attachment method	Submergence time	N	${\boldsymbol{sV}}{{\boldsymbol{O}}}_{{\boldsymbol{2}}}$	$s\dot{{\boldsymbol{V}}}{{\boldsymbol{O}}}_{{\boldsymbol{2}}}$
**Small females and juveniles**											
AFS	AFF1	69–78	17	RF2	G6a+	25 Hz	Tape	1.46 (0.20)	12	45.28 (8.47)	21.01 (2.94)
ASL	ASF4	66	17	RF1	G6a+	25 Hz	Harness	1.43 (0.58)	7	45.72 (27.89)	22.35 (7.04)
ASL	ASF1*	47	5	RF1	G6a+	25 Hz	Harness	1.39 (0.12)	8	56.22 (6.93)	29.43 (3.06)
**NZFS**	NFM1*	54–55	8	RF3	G6a+	25 Hz	Tape	1.03 (0.19)	7	36.13 (9.69)	34.52 (5.74)
**Large males**											
AFS	AFM1	179–182	14	RF2	G6a+	25 Hz	Tape	1.45 (0.36)	24	35.58 (11.26)	17.68 (5.02)
ASL	ASM1	153–160	12	RF3	G6a+	25 Hz	Tape	1.35 (0.32)	17	28.65 (8.43)	15.37 (3.03)
ASL	ASM2	110–125	9	RF1	G6a+	25 Hz	Harness	2.03 (0.31)	7	68.02 (12.67)	16.87 (3.39)
NZFS	NFM2	149–161	11	RF2	G6a+	25 Hz	Tape	2.17 (0.44)	23	50.77 (17.97)	10.64 (1.59)
NZFS	NFM3	154	13	RF3	G6a+	25 Hz	Tape	0.95 (0.10)	15	16.47 (2.58)	18.42 (2.04)
**Large females**											
SSL	F00BO	155–160	15	RF4	Daily Diary	32 Hz	Harness	2.31 (0.75)	33	53.84 (25.55)	10.52 (3.87)
SSL	F97HA	172–175	18	RF4	Daily Diary	32 Hz	Harness	2.19 (0.81)	33	54.58 (27.30)	11.94 (3.46)
SSL	F97SI	230–233	18	RF4	Daily Diary	32 Hz	Harness	2.38 (0.67)	29	53.53 (22.37)	9.49 (1.63)
SSL	F00YA	214–218	15	RF4	Daily Diary	32 Hz	Harness	2.35 (0.85)	35	56.78 (30.23)	10.42 (2.59)

Mean (±SD) and number of trials for $sV{O}_{2}$ (ml kg^−1^) and $s\dot{V}{O}_{2}$ (ml min^−1^ kg^−1^) measured after activity, with time spent submerged (min), species, ID, mass (kg), age (years) and marine facility where housed, type of accelerometer used, recording rate and method of attachment for five fur seals and eight sea lions. Marine facility: RF1 – Dolphin Marine Magic; RF2 – Underwater World; RF3 – Taronga Zoo; RF4 – Open Water Research Station. Species: AFS – Australian fur seal; ASL – Australian sea lion; NZFS – New Zealand fur seal; SSL – Steller sea lion. *Indicates seals identified as subadults during trials.

### Effects of dynamic body acceleration (DBA) calculation methods

**Figure 1:**
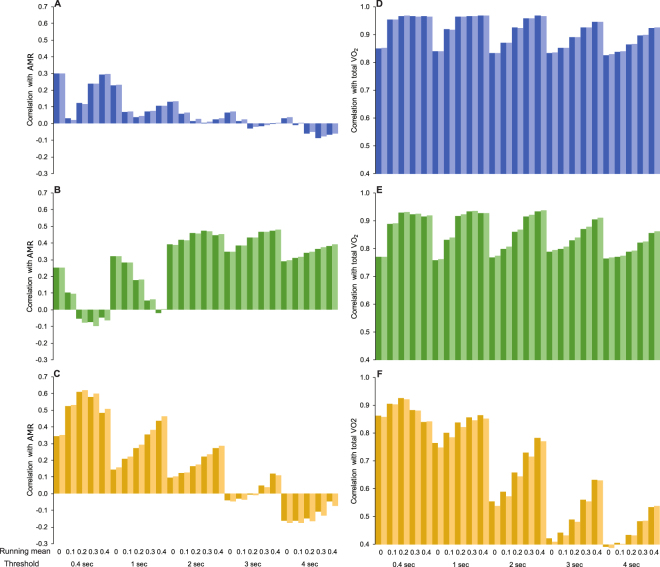
Pearson’s correlation coefficients for relationships between combinations of different running means and thresholds for mean ODBA (light colours) and VeDBA (dark colours) with ${s\dot{{\rm{V}}}O}_{2}$ (ml min^−1^ kg^−1^; **A**–**C**) and for total ODBA (light colours) and VeDBA (dark colours) with sVO_2_ (ml kg^−1^; **D**–**F**). Data presented separately for (**A** and **D**) large females diving (N = 4 animals; n = 130 trials); (**B** and **E**) male fur seals and sea lions swimming transitionally (N = 5 animals; n = 86); **C** and **F** female and juvenile fur seals and sea lions swimming transitionally (N = 4 animals; n = 47 trials).

We tested 25 combinations of different thresholds (0–0.4 g) and running means (0.4–4 sec) to calculate the mean and total area under the curve of both measures of DBA (i.e., ODBA and VeDBA), which were correlated against $s\dot{V}{O}_{2}$ (ml min^−1^ kg^−1^) and $sV{O}_{2}$ (ml kg^−1^), respectively (Fig. 1). We found that different combinations of thresholds and running means greatly influenced the overall correlation of DBA measures with both $s\dot{V}{O}_{2}$ (ml min^−1^ kg^−1^) and $sV{O}_{2}$ (ml kg^−1^) for different groups of animals, but there was very little difference between ODBA or VeDBA. For example, the most effective running mean for predicting $s\dot{V}{O}_{2}$ (ml min^−1^ kg^−1^) from mean ODBA and VeDBA was 0.4 second for both large females (Fig. 1A) and small females and subadults (Fig. 1C), while a 2 or 3 second running mean was best for males (Fig. 1B). Using a threshold did not provide any clear improvement of the correlation of $s\dot{V}{O}_{2}$ with either mean ODBA or VeDBA.

A short running mean (0.4 sec) improved the correlation of total DBA (both total ODBA and VeDBA) with $sV{O}_{2}$ (ml kg^−1^) for small females and subadults. However, the choice of running mean for the large females and males did not have a large effect on the relationship of total DBA with $sV{O}_{2}$ (ml kg^−1^), except that using a running mean of 4 second reduced the correlation (Fig. 1D–E). Unlike for $s\dot{V}{O}_{2}$, for all groups using a threshold consistently improved the relationship of both measures of total DBA with $sV{O}_{2}$ (ml kg^−1^), where the larger threshold corresponded to a higher correlation.

### Predicting energy expenditure

LME’s were used to predict the relationship of $sV{O}_{2}$ with both measures of total DBA (i.e., total ODBA and VeDBA), total number of strokes, and submergence time; and to predict the relationship of $s\dot{V}{O}_{2}$ with both mean DBA variables (i.e. mean ODBA and VeDBA), stroke rate, and submergence time. The models used the combination of running mean and a threshold for ODBA and VeDBA that correlated highest with $s\dot{V}{O}_{2}$ and $sV{O}_{2}$ respectively from each group, (as described in the previous section and listed in Tables 2 and 3). The effect of attachment type and location were tested in each of the models and neither improved the AIC or the variance explained; therefore, they were not considered further. For all combinations of LME’s adding individual as a random effect improved the variance explained (the difference between R^2^ all and R^2^ fixed: Tables 2 and 3).

**Table 2:** Results of linear mixed effects models for total energy expenditure.

Response	Predictor	Group	Equation	R^2^ fixed	R^2^ all	LogLik	AIC
Log of total energy expenditure	Submergence time	Males	−2.29 + log(x)1.30	0.82	0.94	42.11	−76.21
		Females/subadults	−2.27 + log(x)1.33	0.72	0.93	11.62	−15.23
		Large females	1.03 + log(x)0.43	0.67	0.75	101.82	−195.64
	Strokes RMW^a^:4 sec; *m* ^b^:30	Males	−3.12 + log(x)1.33	0.58	0.82	2.13	3.74
	Strokes RMW^a^:4 sec; *m* ^b^:30	Females/subadults	3.05 + log(x)0.03	0.40	0.49	−0.51	9.02
	Stroke RMW^a^:1 sec; *m* ^b^:100	Large females	1.77 + log(x)0.43	0.63	0.71	94.00	−180.00
	VeDBA AUC RM:1 sec; T:0.3	Males	−2.27 +S log(x)1.55	0.89	0.89	23.69	−39.39
	VeDBA AUC RM:0.4 sec; T:0.2	Females/subadults	−0.44 + log(x)1.33	0.91	0.93	22.75	−37.50
	ODBA AUC RM:3 sec; T:0.4	Large females	1.42 + log(x)0.43	0.70	0.75	104.41	−200.81

Relationships presented are between total oxygen consumption (ml kg^−1^) with submergence time (min), dynamic body acceleration (g) or strokes. R^2^ fixed is the amount of variation that is explained by the fixed variables (no random effects) in the model. R^2^ all is the amount of variance that is explained by the fixed variables and the random effects (such as individual animal) in the model. ^a^RMW: running mean window; ^b^ *m:* number of consecutive points before a peak (see Ladds *et al*. 2017^27^ for an explanation).

**Table 3:** Results of linear mixed effects models for rate of energy expenditure.

Response	Predictor	Group	Equation	R^2^ fixed	R^2^ all	LogLik	AIC
Log of rate of energy expenditure	Submergence time	Males	1.66–0.009(x)	0.71	0.87	38.91	−69.83
		Females/subadults	1.02–0.007(x)	0.53	0.66	13.65	−19.30
		Large females	3.47−log(x)0.57	0.59	0.88	99.40	−190.80
	RMW^a^:4 sec; *m* ^b^:30	Males	Not significant	0.03	0.66	1.33	5.35
	RMW^a^:4 sec; *m* ^b^:30	Females/subadults	Not significant	0.03	0.54	8.80	−9.60
	RMW^a^:1 sec; *m* ^b^:100	Large females	Not significant	0.04	0.27	−3.05	14.01
	Mean VeDBA RM:3 sec; T:0.4	Males	Not significant	0	0.64	−2.52	13.03
	Mean VeDBA RM:0.4 sec; T:0.2	Females/subadults	Not significant	0	0.35	5.75	−3.35
	Mean ODBA RM:3 sec; T:0.4	Large females	Not significant	0.02	0.24	−5.01	−18.02

Relationships presented are between the rate of oxygen consumption (ml kg^−1^ min^−1^) with submergence time (min), dynamic body acceleration (g) or strokes. R^2^ fixed is the amount of variation that is explained by the fixed variables (no random effects) in the model. R^2^ all is the amount of variance that is explained by the fixed variables and the random effects in the model. ^a^RMW: running mean window; ^b^ *m:* number of consecutive points before a peak (see Ladds *et al*. 2017 for an explanation).

**Figure 2:**
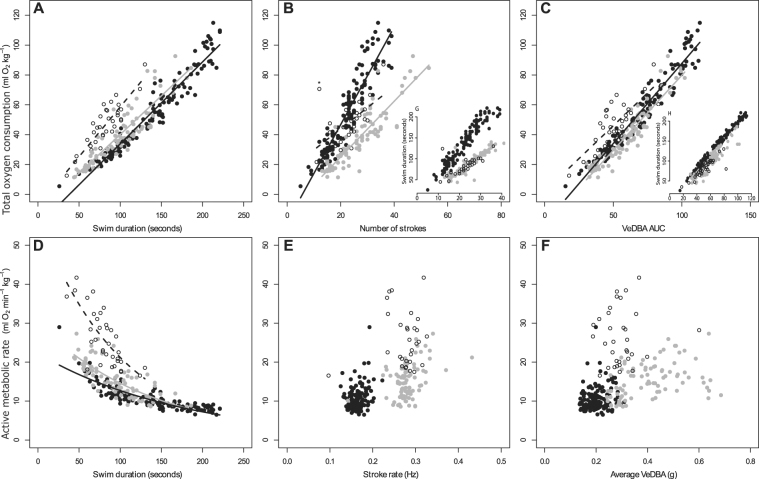
Relationship between total oxygen consumption (${{\rm{sVO}}}_{2}\,\,$ml kg^−1^; top panel) and swim duration (**A**), number of strokes (**B**) and VeDBA AUC (**C**) and relationship between diving metabolic rate (${s\dot{{\rm{V}}}O}_{2}$ ml min^−1^ kg^−1^; bottom panel) and swim duration (**D**), stroke rate (**E**) and average VeDBA (**F**). The relationship between swim duration and of total number of strokes (**G**) and VeDBA AUC (**H**) are displayed for comparative purposes. Open circles are small females and subadults (N = 4 animals; n = 47 trials), closed grey circles are males (N = 5 animals; n = 86) and closed black circles are large females (N = 4 animals; n = 130 trials). For comparisons with other papers the average VeDBA used in F has a running mean of 2 seconds and no threshold^11,36^. *Represents an outlier that was removed when fitting the regression.

**Figure 3:**
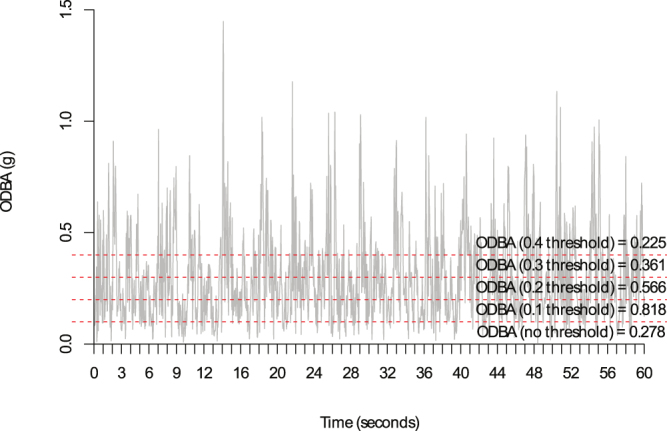
ODBA (g) calculated with a running mean of 2 seconds from a 60 second swim with a comparison of the overall mean ODBA estimated using different thresholds.

For all animal groups $sV{O}_{2}\,$(ml kg^−1^) could be accurately predicted from submergence time (R^2^ fixed = 0.67–82; Fig. 2A–C) with individual accounting for between 8 and 21% additional variation (see R^2^ all, Table 2). Both the total number of strokes and total VeDBA could also predict $sV{O}_{2}\,$(Figs 2B and 3C), but both variables were highly co-linearly related to swim duration (Fig. 2G,H). VeDBA explained more of the variance in $sV{O}_{2}\,$(ml kg^−1^) than submergence time (increased variance explained 3–19%) or total strokes (increased variance explained 7–51%; Table 2).

The strongest predictor of $log\,(s\dot{V}{O}_{2})$ (ml min^−1^ kg^−1^) was submergence time for all groups, reflecting a negative relationship where $\mathrm{log}\,(s\dot{V}{O}_{2})$ (ml min^−1^ kg^−1^) decreased with increased submergence time (Fig. 2D; Table 3). Submergence time alone accounted for 53–59% of the observed variation, while individual animal accounted for an additional 13–29% (Table 3). As predicted, there was no relationship between stroke rate (Hz) and $s\dot{V}{O}_{2}$ (Fig. 2E) or mean VeDBA and $s\dot{V}{O}_{2}$ (Fig. 2F) for any groups.

## Discussion

The relationship between energy expenditure and proxies of body movement, such as stroke rate or measures of DBA (e.g., VeDBA or ODBA) for diving mammals has recently been brought into question^28^. Here we provide evidence using a range of otariids of different ages, sizes, sexes, and species that support Halsey’s contention that many of the strong relationships observed elsewhere between total (summed) energy expenditure and total number of strokes or measures of total DBA are a result of time being incorporated into both sides of the equation. Our study found a strong positive relationship between total energy expenditure (${{\rm{sVO}}}_{2}$ ml kg^−1^), total number of strokes and total DBA (VeDBA or ODBA). However, these measures were also highly collinearly related to submergence time and the apparent relationships observed were partly the result of time correlated with itself.

The effect of time is highlighted further in the observation that measures of mean DBA could not significantly predict rates of energy expenditure. Further, the ability of mean or total DBA (ODBA or VeDBA) to predict energy expenditure (as a rate or a total) changes depending on the running mean and threshold that is used to calculate these measures for different groups of animals. This indicates it is unlikely that a universal equation to estimate the appropriate DBA for a given individual can be derived, further limiting the applicability of this method for estimating energy expenditure in the wild.

### Estimating energy from accelerometers: The time trap

Evidence of a relationship between total flipper strokes and total energy expenditure has been shown in a number of species: Weddell seals^3^, northern elephant seals (*Mirounga angustirostris*)^29^, Antarctic fur seals, and northern fur seals^21^. Similarly, summed VeDBA and total energy expenditure were highly correlated in diving cormorants^24,30^, and for northern fur seals and Antarctic fur seals, albeit only when the energy expenditure was estimated from different relationships for different activities (foraging, transiting, surface movement and resting)^10^. Our study also showed a strong relationship of total energy expenditure ($sV{O}_{2}$ (ml kg^−1^)) with total measures of DBA (total VeDBA and ODBA) and total flipper strokes (Fig. 1B–C). Similar to Antarctic and northern fur seals, we found that total VeDBA was a better predictor of total energy expenditure than total strokes or dive duration, albeit marginally (Table 2)^21^. Submergence time also predicted $\,sV{O}_{2}$ (ml kg^−1^) slightly better than total strokes, contrasting with a study on Weddell seals where the total number of strokes was a better predictor of total energy expenditure than dive duration^3^. This is likely a result of Weddell seals using gliding during a large proportion of their dive, while our otariids stroked relatively constantly throughout each trial with many changes in body orientation. DBA likely incorporated the cost of this additional movement into the model. However, while DBA can account for more of the variance in the relationship due to measuring body movement, most of the variance explained is from the incorporation of time into both the independent and dependent variables. This is demonstrated in our study in the very strong relationships of total VeDBA and total strokes with time (Fig. 1G,H). This so-called “time-trap” means that counting strokes or measuring VeDBA may be no better than simply using the duration an animal spends diving to estimate the cost of that dive^29^.

The effect of the “time trap” is clear when investigating the rate of energy expenditure, that is, by removing time from the equation. When time was removed by expressing the independent ($s\dot{V}{O}_{2}$ (ml min^−1^ kg^−1^)) and dependent variables (mean VeDBA or stroke rate) as rates, no such relationship was evident (Fig. 1D–F). It has been noted that correlations of mean DBA with a rate of energy expenditure in mammal divers may be difficult to establish if oxygen stores were not replenished to the same level between each dive, resulting in inaccurate measures of metabolic rate^31^. We accounted for this by measuring a baseline before each trial and ensuring that metabolic rates returned to within 5% of this value before attempting another trial.

While our study suggests that converting total measures to rates results in the loss of the relationship between energy expenditure and DBA, other studies suggest that the effect of time scale may be species dependent. When measuring average partial DBA (PDBA) and $s\dot{V}{O}_{2}$ in turtles there was no relationship for single dives but a strong relationship was evident for bouts of diving^32^. These results were supported by Halsey *et al*.^33^ 2011) who demonstrated that the relationship between a rate of energy expenditure and ODBA does indeed exist for turtles and offer a good explanation for why this relationship exists in turtles, but not for diving mammals or birds. In cormorants, average daily ODBA and VeDBA correlated with mass-specific daily energy expenditure measured from DLW^30^, but ODBA did not correlate with $s\dot{V}{O}_{2}$ over a single dive cycle^24^. By comparison, when the relationship was examined in otariids there was no relationship between mean ODBA and $s\dot{V}{O}_{2}$ single dives or during bouts of diving^22,23^.

While we have empirically supported the ‘‘time trap’’ hypothesis this does not indicate that measures of dynamic body acceleration have no effect on energy expenditure. We observed that VeDBA accounted for up to 19% more of the variation in measured total oxygen consumption than swim duration alone, and up to 50% more of the variation explained by stroke rate. The inability of mean values of DBA to accurately predict rates of oxygen consumption appears due to either a) some biomechanical aspect of otariid swimming (e.g., large changes in orientation that have little energetic cost), b) some underlying physiological process related to the temporal disconnect between energy expenditure while submerged and post-activity measures of energy expenditure^33^, or c) is a statistical artefact (e.g. insufficient variation in measured DBA and stroke rates). Regardless, our study clearly illustrates the statistical misrepresentation that can occur when using total measures of DBA and energy expenditure, providing support for the “time trap” hypothesis.

### Testing parameters for establishing DBA

When using accelerometers to establish proxies of energy expenditure, the decisions made during the derivation of ODBA or VeDBA affect its predictive ability^34^. This is often an underappreciated source of variation in these techniques. In this study we used two types of DBA: either summing the absolute (ODBA) or taking the square root of the sum (VeDBA) of the dynamic acceleration^13^. Dynamic acceleration is derived from applying a running mean over the axes of acceleration to calculate static acceleration (gravity) and removing this from the raw acceleration^35^. The value used to calculate the running mean changes the value of the DBA, and thus affects the ability of DBA to predict energy expenditure and to calculate an accurate estimate of stroke rate^36^.

Different combinations of the parameters changed the values of the DBA. The large effect of these combinations arose from factors attributable to either the animal or the device. Considering that the males and the large females were roughly the same size during trials, differences were most likely due to sampling frequency and placement of the accelerometer. The accelerometer fitted to large females recorded at 32 Hz and was secured in a harness while the accelerometer fitted to males recorded at 25 Hz and was taped directly to the fur. There was more movement, and thus more signal changes, in the accelerometer on the harness. In the wild, accelerometers are generally attached to fur with glue, thereby reducing the amount of noise in the accelerometry signal. In this experiment, taping the accelerometer to seals more closely resemble this method. Therefore, when extrapolating these results, the combinations of threshold and running mean used for the large male data will likely return the most accurate estimate for VeDBA (as this was a slightly better predictor than ODBA) and number of strokes.

### Conclusions

Measuring the energetic expenditure of free-living marine mammals is fundamental to understanding free ranging behaviour, and in predicting how they will cope with environmental changes. Accelerometers initially showed great promise for measuring energy expenditure over long deployments, but the experimental results of this study demonstrate that accelerometry does not measure the amount of energy expended from an activity, but instead measures the amount of time in that activity. These results suggest that recent accounts of a relationship between DBA and energy expenditure have not demonstrated real relationships, but instead have fallen into the “time-trap”^26^. By using the summed energy expenditure and relating it to a summed proxy, time is being incorperated into both sides of the equation that falsely inflates the apparent underlying relationship. When removing time from the equation by including a rate of energy expenditure, the relationship to all tested proxies disappears. Therefore, while accelerometers may be useful to derive activity budgets from which to estimate energy expenditure^10,37^, it appears unwise to use them to estimate energy expenditure directly.

## Methods

### Animals

We conducted experiments with three New Zealand fur seals (*Arctocephalus forsteri*), two Australian fur seals (*Arctocephalus pusillus*), four Australian sea lions (*Neophoca cinerea*), and four Steller sea lions (*Eumetopias jubatus*) (see Table 1 for details of the animals) at four research facilities: Dolphin Marine Magic (RF1: Coffs Harbour, NSW, Australia); Underwater World (RF2: Mooloolaba, QLD, Australia), Taronga Zoo (RF3: Sydney, NSW, Australia) and Open Water Research Station (RF4: Port Moody, BC, Canada). Experiments were conducted between October and December 2014 at RF1-3 and between November and December 2015 at RF4. All animals were on permanent display or were housed for research purposes, were non-reproductive during the study period and were cared for under the husbandry guidelines of the individual facility. All animals were in good health and condition as assessed by the in-house veterinary surgeon at the time of the experiments. All animal handling and experimental protocols in Australia were specifically authorized by and conducted in accordance with regulations of the Macquarie University ethics committee (ARA-2012_064) and the Taronga ethics committee (4c/10/13), and all experimental protocols were specifically authorized by the Department of Sustainability and Environment Australia. All animal handling and experimental procedures in Canada were conducted in accordance with regulations of the Canadian Council on Animal Care, and all experimental protocols were specifically authorized by the Department of Fisheries and Oceans Canada (License MML 2007-001) and approved by the Animal Care Committees of the University of British Columbia (Permit #A11-0397) and the Vancouver Aquarium.

### Trial protocol and metabolic measurements

During all experiments otariids were equipped with a 3-axis accelerometer (RF1-3: CEFAS G6a+, ±8 g, 40 × 28 × 16.3 mm and mass 18 g in air and 4.3 g in seawater, CEFAS technology Ltd, Lowestoft, UK; RF4: Daily Diary, 95 × 45 × 26 mm, 90 g, Wildlife Computers; Table 1). All sea lions (except ASM2) wore a tight-fitting harness containing the accelerometer while all fur seals (and ASM2) had the accelerometer attached to the fur with tape.

The experimental set-up for collecting oxygen consumption data has been described elsewhere^22,23,38^ so is briefly summarised here. Oxygen consumption was measured before and after subsurface swimming using open-flow respirometry to estimate surface metabolic rate (MRs) and active metabolic rate (AMR). To estimate MRs prior to swimming, otariids would float near motionless under the floating respirometry hood (RF1-3 – 80 L; RF4 – 100 L) until a consistent baseline rate of oxygen consumption was collected for a minimum of 3 min. Prior to trials otariids had not been fed for a minimum of 14 hours (post-absorptive), were resting in husbandry pools, were adult, not pregnant and remained within their assumed thermo-neutral zone during the trials (as determined by water temperature).

To obtain measures of active metabolic rate (AMR), otariids would swim submerged for a pre-determined time before returning to the hood where they remained until their instantaneous rates of oxygen consumption returned to within 5% of levels measured prior to swimming (MRs), ensuring that all dives were independent. The respirometry hood was connected to an open-flow respirometry system (Sable Systems International, Inc., Henderson, NV, USA). Rates of oxygen consumption ($\dot{V}{O}_{2}$) were calculated using equation 4b from Withers^39^ assuming a respiratory quotient of 0.77^40^. To determine the mass-specific total energy expenditure ($sV{O}_{2}$ ml kg^−1^) used during a trial the total amount of oxygen consumed during post-dive that was greater than pre-dive consumption rates was integrated and divided by mass (kg). To obtain a mass-specific rate of energy expenditure ($s\dot{V}{O}_{2}$ ml min^−1^ kg^−1^) $sV{O}_{2}\,\,$was divided by the submerged duration. Only dives that had a recovery period of longer than 120 seconds were kept for analysis. To estimate AMR, sea lions at RF4 dove to 10 m where they received small pieces (~20 g) of herring at a 5 or 10 second rate while swimming between two submerged feeding stations between 1 and 3 m below the water’s surface^41^, while otariids at RF1-3 were trained to swim laps of a pool between two stationary targets^7^. All animals were familiar with the experimental equipment and performed all trials voluntarily under trainer control. Submergence durations were timed *in situ* at all facilities. The distance covered and submergence time of trials for otariids differed due to differences in experimental set-up, training differences, and motivation of the seal. Some trials were incomplete due to the seal surfacing outside of the hood and were excluded from analysis.

Mass (±2 kg) was recorded once per week of trials for otariids housed at RF1, RF2 and RF3 as a part of their normal routine and at RF4 sea lion mass (±0.5 kg) was measured daily. Animals were delineated into three separate groups for analysis based on their development and sex as these were previously shown to affect metabolic rate, whereas species did not^7^. The resulting groups were small females and juveniles, adult males, and large females (Steller sea lions) (Table 1). The large females were separated from males as they were significantly larger and had a different experimental set-up to the other animals.

### Accelerometer measurements

Accelerometers (described above) recorded time, depth, and acceleration on three axes: anterior-posterior (surge, x-axis), lateral (sway, y-axis) and dorso-ventral (heave, z-axis), from which ODBA, VeDBA and flipper stroke frequency during dives were extracted. To determine stroke frequency from the accelerometry a validation trial was run. We videoed a sub-sample of swims during trials using an underwater camera (GoPro) from which stroke frequency was counted. The raw acceleration was first smoothed using a running mean of three seconds and then time-matched to the video. Strokes were identified as corresponding to peaks in the smoothed acceleration. To automatically determine stroke rate from accelerometers the peaks in the x-axis were identified using a simple function in R that identified a peak from a minimum number of consecutive positive data points. The number of strokes estimated from the peak analysis of the accelerometry data were validated with observed counts from the video analysis.

Both the estimate for overall dynamic body acceleration (ODBA, g) and vectorial dynamic body acceleration (VeDBA, g) change with the running mean selected^36^. We chose to test a range of running means and evaluated how this affected the overall relationship with oxygen consumption. As $\,sV{O}_{2}$ (ml kg^−1^) and $s\dot{V}{O}_{2}$ (ml min^−1^ kg^−1^) only accounts for the energy that is expended above resting, it is theoretically possible to remove the passive component of movement within a swim/dive cycle (where we assume the seal is using their resting metabolism) by removing a threshold (baseline) value. This threshold could potentially account for any of the movement of the accelerometer that was not due to the explicit movement of the animal (i.e., movement of the harness during gliding). Therefore, we also tested the effects of incorporating thresholds of 0, 0.1, 0.2, 0.3 and 0.4 g on predictive capacity (Fig. 3).

We calculated static acceleration for ODBA and VeDBA from each axis using a range of running means: 0.4, 1, 2 and 3 seconds. An estimate of dynamic acceleration was then obtained by subtracting the static acceleration from the raw values. Then, to calculate ODBA the absolute values of each of the dynamic estimates were summed (Eq. 1) and to calculate VeDBA the square root of the summed dynamic estimates is calculated (Eq. 2).

1 $$ODBA=|{X}_{dyn}|+|{Y}_{dyn}|+|{Z}_{dyn}|$$

2 $$VeDBA=\sqrt{{X}_{dyn}^{2}+{Y}_{dyn}^{2}+{Z}_{dyn}^{2}}$$

### Statistical analysis

ODBA and VeDBA mean and area under the curve (AUC) were calculated for every combination of running means of 0.4, 1, 2, 3 or 4 seconds and thresholds of 0, 0.1, 0.2, 0.3 or 0.4 g. In total, there were 100 measures created for DBA (ODBA and VeDBA, both mean and AUC) from combinations of running means and thresholds. Pearson’s correlation coefficient was used to select the variables that demonstrated the strongest correlations with $sV{O}_{2}$ and $s\dot{V}{O}_{2}$ that were then used in the models.

We used multiple linear mixed-effects models (LME) with restricted maximum likelihood (REML) estimation to evaluate which source of variation best explained changes in $sV{O}_{2}$ (ml kg^−1^) and $s\dot{V}{O}_{2}$ (ml min^−1^ kg^−1^) using the NLME package in R^42^. Using $sV{O}_{2}$ and $s\dot{V}{O}_{2}$ as the response variables, we first ran null models (no random effects) to find a baseline from which we could evaluate the influence of the random effect on the models. We then ran LME’s with individual animal as the random effect to account for repeated measures. The predictor variables for the $sV{O}_{2}$ model were: submergence duration, total strokes and VeDBA or ODBA AUC. The predictor variables for the $s\dot{V}{O}_{2}$ model were: submergence duration, stroke rate and VeDBA or ODBA mean. All the predictor variables were tested with species, sex and attachment method as co-variates to determine their influence on the models. The best combination of variables were tested using the function *dredge* from the package MuMln in R.

Model selection was based on a combination of Akaike Information Criteria (AICc), log likelihoods (logLik) and R^2^. The amount of variance explained by the random effect was assessed through the difference of the marginal (fixed effect only) and conditional (all model variables) R^2^ (rsquared.glmm function). The assumptions of homoscedasticity, normality, homogeneity and independence were investigated by plotting predicted versus fitted residuals, QQ-plots, Cleveland dot-plots and ACF plots^43^. Where models did not meet assumptions, we log transformed the predictor and/or the independent variable. All analysis was completed in R Version 3.1.3^44^ and values are reported as mean ± SD. The datasets generated during and analysed during the current study are available in the “Proxies_EnergyExpenditure” repository: https://github.com/MoniqueLadds/Proxies_EnergyExpenditure.git.

### Data availability

Supporting datasets are available in the GitHub “Proxies_EnergyExpenditure” repository: https://github.com/MoniqueLadds/Proxies_EnergyExpenditure.git.
